# Bloodstream infections in patients with malignancies: implications for antibiotic treatment in a Ghanaian tertiary setting

**DOI:** 10.1186/s13104-015-1701-z

**Published:** 2015-12-01

**Authors:** Noah Obeng-Nkrumah, Appiah-Korang Labi, Michael Ebo Acquah, Eric S. Donkor

**Affiliations:** Department of Microbiology, School of Biomedical and Allied Health Sciences, University of Ghana, P.O. Box 4326, Accra, Ghana, West Africa; Department of Microbiology, Korle-Bu Teaching Hospital, P.O. Box 77, Accra, Ghana, West Africa; Department of Haematology, Korle-Bu Teaching Hospital, P.O. Box 77, Accra, Ghana, West Africa

**Keywords:** Ghana, Cancer, Blood stream, Infections, Bacteria, Fungi, Multidrug, Resistant

## Abstract

**Background:**

Bloodstream infections (BSI) remain a major cause of mortality in patients with malignancies. We present the first report on the microbiological profile of bacteraemia and fungaemia among cancer patients in Ghana.

**Methods:**

From January 2010 through December 2013, we retrospectively analyzed the spectrum of bloodstream pathogens in cancer patients from Korle-Bu Teaching Hospital, Ghana—focusing on multidrug resistant isolates (MDRs).

**Results:**

Overall BSI were confirmed in 22 % (n = 93/453) of total blood cultures. Our data highlights a co-dominance of Gram-negative (n = 49/93, 52.6 %) and Gram-positive (n = 40/93, 43.0 %) bacteria with the former less likely to infect children than adults [odds ratio (OR), 0.56; 95 % confidence interval (CI) 0.14–0.91; *p* value = 0.027]. Staphylococcus epidermidis was the most isolated bacteria (30.1 %; n = 28/93). About 61 % (n = 25/41) of *Enterobacteriaceae* isolates were resistant to cefotaxime; a majority (n = 24/25, 96 %) of which were MDRs and mostly susceptible to amikacin and levofloxacin. Four (80 %) penicillin resistant streptococci were found; 2 of which were MDRs and sensitive to erythromycin and cefuroxime. Methicillin resistant *Staphylococcus aureus* and vancomycin resistant enterococci were not identified. In multivariate analysis, the *Enterobacteriaceae* compared to other organisms were significantly associated with multidrug resistance (adjusted OR, 33.6; 95 % CI 6.41–88.73; p value 0.001).

**Conclusion:**

MDRs, especially cefotaxime resistant *Enterobacteriaceae*, are common among patients with cancer in our institution but vary among different patient populations. The results show that empiric antibiotic treatment for cancer patients cannot be done effectively without regard for selective antimicrobial use based on local epidemiologic data.

## Background

Bloodstream infections (BSI) remain a major cause of life-threatening complications in patients with cancer [1]; and are directly associated with prolonged hospital stay, high healthcare costs and increased risk of morbidity and mortality [2, 3]. Overall mortality rate as a result of BSI in cancer patients is reported at 25–32 % [1]. Bacteria are the most common cause [4–6]. Fungi have also been implicated as important aetiologic agents [7]. Surveillance data worldwide on BSI in cancer patients remains limited, and is more so exemplified by the lack of data from Africa especially in western Africa [1, 8]. In Ghana, a wide range of bacterial isolates have been identified in infections at teaching and regional hospitals—with widespread prevalence of multidrug resistance among the various isolates [9, 10]. Although cancer patients are managed in our institution, there are no local data on the causes of BSI within this patient group. In order to adapt policies and encourage a more selective management strategy of cancer patients in Ghana, there is the need for local monitoring of bacterial isolates and antibiogram [11, 12].

In this study, we retrospectively examined the microbiological profile of bacteraemia and fungaemia, over a 4-year period, in patients with malignancies in a tertiary hospital setting in Ghana. There are relatively few microbiologic data on pediatric patients with malignancies reported to date; and available studies on antibiotic resistance associated with cancer patients are often limited to particular antibiotics [13, 14] or to resistant organisms, such as methicillin-resistant *Staphylococcus aureus* [15]. Therefore, our primary outcome was to evaluate the spectrum of bloodstream isolates and report on the antimicrobial susceptibilities of causative pathogens in paediatric and adult cancer patients, focusing on the presence of multidrug resistant phenotypes as a whole.

## Methods

### Study setting

This study was retrospectively conducted at the Korle-Bu Teaching Hospital (KBTH), which is a 2000-bed hospital in Ghana, West Africa. The hospital is the premier referral center in the country and covers all medical specialties. In this study, cancer patients with BSI were identified from the Microbiology Department of KBTH, which processes over 40,000 clinical cultures annually. Three major units handle cancer patients in KBTH—haematology, paediatric oncology and the radio oncology unit.

### Study design and sampling

Between January 2010 and December 2013, we retrospectively studied bloodstream isolates of cancer patients collected at the bacteriology unit of the Central Microbiology Laboratory in KBTH. Two sampling approaches were used. First, all isolates recovered from blood cultures of cancer patients submitted to the microbiology laboratory of KBTH were characterized and analysed. Second, we reviewed laboratory records of all cancer patients with BSI. Blood culture isolates were selected on the following criteria: (1) confirmed as the causative agent of the infection for which blood cultures were performed, and (2) identified as first isolate per patient within the study period. Multiple isolates of the same bacteria per patient were only considered if antibiotic susceptibility patterns were different, and patients’ bacteraemia episodes were more than 2 months apart.

### Culture and identification

For each patient, 1–3 mL (for paediatric patients) and 8–10 mL (for adults) of blood cultures injected directly into Bactec^®^ culture vials (Becton–Dickinson, USA) were submitted for laboratory diagnosis. Blood cultures were processed with Bactec^®^ 9240 blood culture system (Becton–Dickinson, NJ, USA) according to manufacturer’s instructions. The presence of BSI was defined by at least one set of positive blood culture for bacteria or fungi in patients. When blood cultures yielded positive results, they were evaluated to determine whether it represented true bacteraemia, fungaemia, or simply contamination. For potential skin contaminants (e.g., micrococci, *Staphylococcus epidermidis*), at least two separate positive blood cultures obtained from different sites had to be isolated for the results to be considered as true infection. A total of 111 blood cultures from cancer patients were positive for various organisms. Aerobic subcultures were made from positive culture vials onto blood, chocolate, MacConkey and Saboraund agar. Identification of bacteria were carried out by Gram-stain microscopy and by conventional biochemical methods. Species identity were confirmed by the BBL crystal identification system (Becton–Dickinson, NJ, USA). Positive blood cultures for yeast and non-yeast fungi were identified on the basis of morphology.

### Susceptibility testing

The susceptibilities of Gram-negative bacteria (GNB) to the antimicrobial agents ampicillin (10 µg) amoxicillin/clavulanate (30 µg), cefuroxime (30 µg), cefotaxime (30 µg), chloramphenicol (30 µg), cotrimoxazole (25 µg), gentamicin (10 µg), amikacin (30 µg), ciprofloxacin (5 µg), tetracycline (30 µg) and levofloxacin (5 µg) (HiMedia Laboratories, India) were determined with Kirby-Bauer disc diffusion method in accordance with the Clinical and Laboratory Standards Institute (CLSI) guidelines [16]. Erythromycin (30 µg), oxacillin (30 µg), and vancomycin (30 µg) were included for Gram positive bacteria (GPB). The reference strain *E. coli* ATCC 25922 and *Staphylococcus aureus* ATCC 10221 were included as quality controls in the susceptibility assays. Relative to the panel of antibiotics tested for each isolate, and according to the international standard definitions for acquired resistance, multidrug resistant (MDR) phenotype was defined as in vitro non-susceptibility to ≥1 agent in ≥3 antimicrobial categories [17]: penicillins, cephalosporins, beta-lactamase inhibitor combinations, fluoroquinolones, aminoglycosides, chloramphenicol, folate pathway inhibitors, tetracyclines, macrolides and glycopeptides.

### Patients’ record review

To provide accurate information, patients and isolates data were abstracted in the following two steps, (1) manual work through of laboratory records, and (2) physician-assisted medical reconciliation of laboratory data. Positive cultures were categorized into GNB and GPB. Data were retrospectively reviewed and compared. Study data was compared between the following: cancer patients with Gram-positive BSI and those with Gram-negative BSI; cancer patients with MDR BSI and those with non-MDR BSI; as well as study patients infected with cefotaxime resistant isolates and those with cefotaxime susceptible strains. Each patient was included only once for each outcome. Patients groups were compared regarding the following: demographics (age, gender), year of infection as well as assigned department of care. The data also included variables relating to infecting isolates, presence of febrile neutropenia, and cancer types. Paediatric patients were defined as patients aged 0–13 years; adults were those >13 years old.

### Statistics

Study data was captured into Microsoft Excel, and exported into Statistical Package for Social Sciences (SPSS, version 20.0) for editing and statistical analyses. Where appropriate, data were compared between two consecutive study periods (Period 1, 2010 through 2011; Period 2, 2012 through 2013) so that trends could be ascertained. Comparisons between categorical data were conducted with Fisher’s exact test or Chi-square with Marascuilo’s post hoc tests for multiple comparisons. Changes in incidence over time were analyzed by Chi-square test for trend. Continuous variables are expressed as mean ± standard deviation. Normalized continuous data were compared using students’ t test with analyses of variance (ANOVA) for multiple comparisons. The Mann–Whitney U test was used for non-parametric continuous distributions. Point estimates of statistical significance were indicated with 2-tailed p values <0.05. Univariate analysis were computed with odds ratio (OR) with 95 % confidence interval (CI); variables with a p value <0.05 were analyzed in multivariate logistic regression models to identify independently associated predictor variable(s). Predictive accuracy of the models was evaluated by Hosmer and Lemeshow goodness-of-fit test with p value >0.05 suggesting that the model predicts accurately on average.

### Ethical considerations

The bacteriology unit the Microbiology Department of KBTH regularly de-identifies patients and their laboratory records. The authors were not privy to patients’ laboratory data prior to anonymization. On receipt of de-identified patients’ laboratory data and isolates, we additionally anonymized all patients to ensure complete obscurity from laboratory archives. We further allotted arbitrary numbers to all isolates assigned to the study. Considering the anonymized data, as well as the retrospective nature of the study, we could not obtain patients’ consent for use of the laboratory records. Patients’ clinical notes were not reviewed for further information. Ethical approval for isolates and laboratory data was not required as the study was regarded as part of routine surveillance measures for infection control.

## Results

### General characteristics

During the study period, and as part of patient management, 23,708 blood cultures were submitted for microbiological investigations. Overall, 453 of the 23,708 blood cultures were received from cancer patients from haematology, paediatric oncology and other allied units for laboratory investigations. Excluding negative results (n = 341/453; 75.4 %) and contaminants [(n = 18/453, 3.9 %)—mostly *Bacillus* species (n = 9/18, 50 %)], a total of 93 episodes of BSI were observed (Table 1). From these, 18 (19.4 %) isolates were recovered in 2010, 29 (31.1 %) in 2011, 31 (33.3 %) in 2012, and 11 (11.8 %) in 2013. About 6.4 % (n = 11/93) of the BSI were polymicrobial—mostly *Staphylococcusepidemidis* plus *Escherichiacoli* (63.6 %, n = 7/11). The median age was 34.0 years (range 1 year–78 years). More than 63 % (n = 59/93) of cancer patients with BSI were children. Males comprised 60 % (n = 55/93) of the patients. Overall, 54.8 % (n = 54/93) of BSIs occurred in patients with hematological malignancies.

**Table 1:** Causative organisms of all episodes of bacteraemia compared by study periods

Isolates	Period 1			Period 2			p value for X^2^ trend
	Total	2010 (n = 18)	2011 (n = 30)	Total	2012 (n = 33)	2013 (n = 12)	
Gram negatives (n = 49)	^a^25	^a^11_a_	^a^14_a_	^a^24	^a^19_a_	^a^5_a_	0.504
*Enterobacteriaceae* (n = 32)	16	8_a_	8_a_	16	12_a_	4_a_	0.658
*Escherichia coli* (n = 8)	5	3	2	3	1	2	0.469
*Klebsiella pneumoniae* (n = 3)	0	0	0	3	3	0	0.442
*Citrobacter* species (n = 12)	8	4	4	4	4	0	0.071
*Enterobacter* species (n = 7)	1	0	1	6	4	2	0.058
*Providencia* specie*s* (n = 1)	1	0	1	0	0	0	0.329
*Salmonella* typhimurium (n = 1)	1	1	0	0	0	0	0.045
*Acinetobacter* species (n = 7)	3	3	0	4	4	0	0.314
*Pseudomonas aeruginosa* (n = 10)	6	0	6	4	3	1	0.896
Gram positives (n = 40)	^a^22	^b^7_a_	^a^15_a_	18^a^	^a^12_a_	^a^6_a_	0.896
*Enterococcus* species (n = 2)	1	1	0	1	1	0	0.881
*Staphylococcus* aureus (n = 4)	3	1	2	1	1	0	0.241
*Staphylococcus* *epidermidis* (n = 28)	16	5	11	12	8	4	0.738
*Streptococcus* species (n = 6)	2	0	2	4	2	2	0.184
Fungi (n = 4)							
*Candida albicans* (n = 4)	^b^1	^b^0_a_	^b^1_a_	^b^3_a_	^b^2_a_	^b^1_a_	0.325

Numbers with differing superscripts and subscripts within columns and rows, respectively, are significantly different at the p < 0.05

*X* ^*2*^ Chi-square linear trend test with Mantel–Haenszel statistic, *Period 1* first study period, *Period 2* second study period

### Causative organisms

**Fig. 1:**
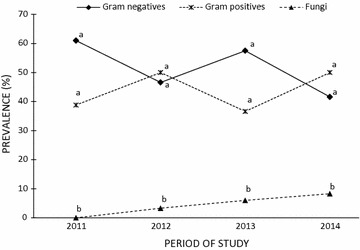
Aetiology of bloodstream infections in cancer patients at Korle-Bu Teaching Hospital from 2010 to 2013. Differing superscripts within years are significantly different at the p value <0.05. No significant difference in prevalence of Gram-positive, and Gram-negative organisms was noted within the years of study. For each year, the number of fungi recovered was significantly (p value <0.05) less compared to Gram-positive, and Gram-negative bacteria. Changes in incidence of Gram negatives, Gram positives, or fungi over the 4-year period were not significant (p value >0.05 for each group, Chi-square for trend analysis)

Of the 93 patients with bloodstream infections (Table 1), 40 (43.0 %) had Gram positive bacteria (mostly *Staphylococcus epidermidis*, n = 28), 49 (52.6 %) had Gram-negative bacteria (*Enterobacteriaceae*, n = 32; *Pseudomonas aeruginosa*, n = 10; *Acinetobacter* species, n = 7), and 4 (4.3 %) were fungi (all *Candida* species). Chi square trend for analysis showed that the proportions of GNB (p value for X^2^ trend = 0.504), GPB (p value for X^2^ trend = 0.896) and fungi (p value for X^2^ trend = 0.325) in BSI of cancer patients did not significantly change over the study period (Fig. 1).

### Distribution of blood culture isolates

Table 2 shows the distribution of GPB and GNB across patients’ groups. Over the 4-year period, the proportion of blood cultures positive in adults (20.4 %; n = 34/167) and paediatric patients (21.5 %; n = 59/274) were similar (p value = 0.769). Also, the proportion of BSI due to GPB was greater (p value = 0.028) in children (52.5 %, n = 31/59) than in adults (26.5 %, n = 9/34). The GNB less often caused BSI in paediatric patients than adults (OR 0.56; 95 % CI 0.14–0.91; p value = 0.027). Overall, the proportion of BSIs between pediatric and adult populations did not differ for any bacterial species except *E. coli* which caused more (p value = 0.018) BSI in adults (n = 6/34, 17.6 %) than children (n = 2/59, 3.3 %). There was no difference in the proportion of GPB or GNB between patients with haematologic cancers and those with non-haematologic malignancies.

**Table 2:** Distribution of blood culture isolates across patients’ groups

Microorganisms	Patients’ group (%)			Cancers				p value	Febrile neutropenia (n = 23)
				Haematologic			Non-haematol. (n = 39)		
	Paeds. (n = 59)	Adults (n = 34)	p value	Leuk. (n = 35)	Lymp. (n = 15)	Myel. (n = 4)			
Gram negatives (n = 49)	^a^26	^a^23	0.028	^a^22 (62.8)_a_	^a^9 (60.0)_a_	^a^2 (50.0)_a_	^a^16 (41.0)_a_	0.056	^a^9
*Enterobacteriaceae* (n = 32)	16	16	0.051	15	8	1	12	0.182	6
*Escherichia coli* (n = 8)	2	6	0.018	5	0_a_	1	2	0.310	1
*K. pneumoniae* (n = 3)	2	1	0.906	1	0	0	2	0.378	2
*Citrobacter* species (n = 12)	6	6	0.300	6	2	0_a_	4	0.517	1
*Enterobacter* species (n = 7)	5	2	0.648	2	2	0	3	0.959	2
*Providencia* species (n = 1)	1	0	0.455	0	1	0	0	0.393	0
*Salmonella* typhimurium (n = 1)	1	0	0.455	0	0	0	1	0.236	0
*Acinetobacter* *species* (n = 7)	5	2	0.648	1	3	1	2	0.456	2
*Pseudomonas aeruginosa* (n = 10)	5	5	0.350	7	1	0	2	0.137	1
Gram positives (n = 40)	^a^31	^b^9	0.014	^b^10 (28.5)_a_	^a^6 (40.0)_a_	^a^2 (50.0)_a_	^a^21 (53.8)_a_	0.048	^a^12
*Enterococcus* species (n = 2)	2	0	0.278	0	0	0	2	0.093	1
*Staphylococcus* *aureus* (n = 4)	3	1	0.623	0	1	1	2	0.738	0
*Staphylococcus epidermidis* (n = 28)	21	7	0.128	10	2	0	15	0.088	9
*Streptococcus* species (n = 6)	5	1	0.295	0	3	1	2	0.659	2
Fungi (n = 4)									
*Candida* species	^b^2	^c^2	0.568	2 (5.7)^c^	^b^0	^a^0	^b^2 (5.3)	0.738	^b^2

Numbers with differing superscripts within columns or differing subscripts within rows are significantly different at the p < 0.05

*Paeds* paediatric patients, *Leuk*. leukemia, *Lymph*. lymphoma, *Myel* myeloma, *Non-haematol*. non-haematologic malignancies (type not confirmed)

### Antibiotic susceptibility

In Table 3, differences in antibiogram between 49 Gram-negative and 40 Gram-positive bacteria recovered from blood cultures of cancer patients are shown. Four (80 %) of the five GNB tested against amoxicillin/clavulanate were resistant and about one-half (excluding *Pseudomonas* species) were resistant to cefotaxime (60.9 %, n = 25/41). Greater than 60 % of the GNB were also susceptible to gentamicin (60.5 %, n = 24/39), ciprofloxacin (61.2 %, n = 11/18) and amikacin (63.8 %, n = 25/38). Overall, levofloxacin was the most effective antibiotic against GNB (76.5 %, n = 13/17). Regarding GPB, few displayed resistance to gentamicin (16.7 %, n = 6/36) and ampicillin (27.7 %, n = 10/37). Most GPB were susceptible to erythromycin (91.2 %, n = 31/34) and vancomycin (87.5 %, n = 28/32).

**Table 3:** Antibiotic susceptibility of blood culture isolates from cancer patients at KBTH

Microorganisms	Resistance to antimicrobial agents (%)										
	AMP	CRX	AUG	GEN	AMK	CIP	LEV	COT	TET	CTX	CHL
Gram negatives (n = 49)	^a^33/33 (100.0)	^a^28/36 (77.7)	^a^4/5 (80.0)	^a^15/39 (39.5)	13/38 (34.2)	7/18 (38.8)	4/17 (23.5)	^a^30/33 (90.9)	^a^28/30 (93.3)	25/41 (60.9)	25/29 (86.2)
*Enterobacteriaceae* (n = 32)	28/28 (100.0)	23/29 (79.3)	3/4 (75.0)	9/24 (42.8)	9/31 (29.0)	7/11 (63.6)	2/8 (40.0)	25/28 (89.9)	24/25 (96.0)	20/32 (62.5)	20/23 (86.9)
*Escherichia coli* (n = 8)	7/7 (100.0)	4/5 (80.0)	1/1 (100)	3/8 (37.5)	4/7 (57.1)	4/5 (80.0)	0/2 (0.0)	6/6 (100)	5/6 (83.3)	4/8 (50.0)	6/6 (100)
*K. pneumoniae* (n = 3)	3/3 (100.0)	2/3 (66.7)	–	2/3 (66.7)	0/3 (0.0)	1/1 (100)	0/1 (0.0)	3/3 (100)	3/3 (100)	3/3 (100)	3/3 (100)
*Citrobacter* species (n = 12)	9/9 (100.0)	9/12 (75.0)	0/1 (0.0)	4/12 (33.3)	3/12 (25.0)	0/1 (0.0)	1/3 (33.3)	8/11 (72.7)	10/10 (100)	7/12 (63.6)	7/10 (70.0)
*Enterobacter* species (n = 7)	7/7 (100.0)	7/7 (100.0)	2/2 (100.0)	–	2/7 (28.7)	2/4 (50.0)	1/2 (50.0)	6/6 (100)	5/5 (100)	6/7 (85.7)	4/4 (100)
*Providencia* species (n = 1)	1/1 (100)	0/1 (0.0)	–	0/1 (0.0)	0/1 (0.0)	–	–	1/1 (100)	–	0/1 (0.0)	–
*Salmonella* typhimurium (n = 1)	1/1 (100)	1/1 (100)	–	–	0/1 (0.0)	–	–	1/1 (100)	1/1 (100)	0/1 (0.0)	–
*Acinetobacter* *species* (n = 7)	5/5 (100.0)	5/7 (71.4)	1/1 (100)	6/7 (85.7)	4/7 (57.1)	0/1 (0.0)	2/3 (66.7)	5/5 (100)	5/5 (100)	5/7 (71.4)	5/6 (83.3)
*Pseudomonas aeruginosa* (n = 10)	–	–	–	0/8 (0.0)	–	0/6 (0.0)	0/6 (0.0)	_–_	–	–	–

	AMP	CRX	AUG	GEN	ERY	CLO	VAN	COT	TET
Gram positives (n = 40)	^b^10/37 (27.7)	^b^10/39 (25.6)	^a^2/3 (66.7)	^b^6/36 (16.7)	3/34 (8.8)	0/4 (0.0)	4/32 (12.5)	^b^2/12 (16.6)	^b^2/5 (40.0)
*Enterococcus* species (n = 2)	1/1 (100.0)	1/1 (100.0)	–	2/2 (100.0)	1/1 (100)	–	0/1 (0.0)	–	0/1 (0.0)
*Staphylococcus* *aureus* (n = 4)	3/3 (100.0)	2/4 (50.0)	0/1 (0.0)	0/4 (0.0)	1/3 (33.3)	0/4 (0.0)	0/1 (0.0)	1/1 (100)	2/3 (66.7)
*Staphylococcus epidermidis* (n = 28)	2/28 (7.1)	2/28 (7.1)	–	2/28 (7.1)	1/28 (3.5)	–	2/28 (7.1)	1/10 (10.0)	0/1 (0.0)
*Streptococcus* species (n = 6)	4/5 (80.0)	5/6 (83.3)	2/2 (100)	2/2 (100.0)	0/2 (0.0)	–	2/2 (100)	–	–

Numbers with differing superscripts between Gram-positives and Gram negatives within an antimicrobial agent are significantly different at the p < 0.05

*AMP* ampicillin, *CRX* cefuroxime, *AUG* amoxicillin/clavulanate, *GEN* gentamicin, *AMK* amikacin, *CIP* ciprofloxacin, *LEV* levofloxacin, *COT* cotrimoxazole, *TET* tetracycline, *CTX* cefotaxime, *CHL* chloramphenicol

### Multidrug resistance

In this study, 44.9 % (n = 40/89) of the bacterial isolates recovered from blood cultures of cancer patients were multidrug resistant (Table 4). The MDR phenotypes were significantly less in GPB (n = 6/40, 15.0 %) compared to GNB (n = 34/49, 69.3 %). Focusing on specific MDR pathogens of worldwide interest, in vitro non-susceptibility to cefotaxime (30 µg) was used as a presumptive screen positive test for third generation cephalosporin resistant *Enterobacteriaceae* [16]. About 61 % (n = 25/41) of *Enterobacteriaceae* isolates were cefotaxime resistant. A significant majority (n = 24/25, 96 %) of these were MDRs. Among the Gram positives, multidrug resistant phenotypes were reported in *Enterococcus* species (n = 1/2, 50 %), *Staphylococcus aureus* (n = 1/4, 25 %), *Staphylococcus epidermidis* (n = 2/28, 25 %) and *Streptococcus* species (n = 2/6, 33.3 %). All the MDR *S. epidermidis* were resistant to vancomycin but susceptible to erythromycin. This study did not identify any methicillin resistant *S. aureus* (based on oxacillin susceptibility) or vancomycin resistant enterococci. Based on ampicillin resistance, four (80 %) penicillin resistant streptococci were found; 2 of which were vancomycin resistant MDRs.

**Table 4:** Multiple drug resistant strains recovered from blood cultures of cancer patients

Microorganism	Multiple drug resistant isolates (%)								
	Total	Period 1				Period 2			
		Number	CTX-R	CTX-S	p value	Number	CTX-R	CTX-S	p value
Gram negatives (n = 49)	^a^34/49 (69.3)	^a^15/25_a_ (60.0)	9/15	6/15	0.273	^a^19/24_a_ (79.2)	16/19	3/19	<0.001
*Enterobacteriaceae* (n = 32)	28/32 (87.5)	12/16_a_ (75.0)	7/12	5/12	0.414	16/16_b_ (100.0)	13/16	3/16	<0.001
*Escherichia coli* (n = 8)	8/8 (100)	5/5_a_ (100)	3/5	2/5	0.527	3/3_a_ (100)	1/3	2/3	0.414
*K. pneumoniae* (n = 3)	3/3 (100)	0	0	0	–	3/3 (100)	3/3	0/3	0.014
*Citrobacter* species (n = 12)	9/12 (75.0)	5/8_a_ (62.5)	3/5	2/5	0.527	4/4_a_ (100)	4/4	0/4	0.004
*Enterobacter* species (n = 7)	7/7 (100)	1/1_a_ (100)	1/1	0/1	–	5/6_a_ (100)	5/6	0/6	<0.001
*Providencia* species (n = 1)	0/1 (0.0)	0/1	0	0	–	0	0	0	–
*Salmonella* typhimurium (n = 1)	1/1 (100)	1/1 (100)	0/1	1/1	–	0	0	0	–
*Acinetobacter* *species* (n = 7)	6/7 (85.7)	3/3_a_ (100)	2/3	1/3	0.414	3/4_a_ (75)	3/4	0/4	0.028
*Pseudomonas aeruginosa* (n = 10)	0/10 (0.0)	0/6_a_ (0.0)	–	–		0/4_a_ (0.0)	–	–	
Gram positives (n = 40)	^b^6/40 (15.0)	^b^1/22_a_ (4.5)	–	–		^b^5/18_b_	–	–	
*Enterococcus* species (n = 2)	1/2 (50.0)	1/1_a_ (50.0)	–	–		0/1_a_ (0.0)	–	–	
*Staphylococcus* *aureus* (n = 4)	1/4 (25.0)	0/3_a_ (0.0)	–	–		1/1_a_ (100)	–	–	
*Staphylococcus epidermidis* (n = 28)	2/28 (7.2)	0/16_a_ (0.0)	–	–		2/12_a_ (16.7)	–	–	
*Streptococcus* species (n = 6)	2/6 (33.3)	1/2_a_ (50.0)	–	–		1/4_a_ (25.0)	–	–	
Total	39/89 (43.9)	16/47_a_ (34.1)				23/42_b_ (54.7)			

Numbers with differing superscripts within antimicrobial agent are significantly different at the p < 0.05

*CTX-R* cefotaxime (30 μg) resistant, *CTX-S* cefotaxime susceptible, *Period 1* first study period, *Period 2* second study period

### Characteristics of patients with MDR BSI

Table 5 shows the characteristics of cancer patients with BSI caused by MDR and non-MDR bacteria. Children were less likely to be infected with MDR organisms compared to adults. Patients with haematologic malignancies compared to those with non-haematologic cancers more frequently had MDR BSI. Compared to non-MDRs, MDR isolates were more frequently *Enterobacteriaceae*. In multivariate analysis, the *Enterobacteriaceae* compared to other bacteria were significantly associated with MDR after adjusting for age, paediatric patients, cefotaxime resistance, Gram-negative bacteria and Pseudomonads (adjusted odds ratio 33.6; 95 % CI 6.41–88.73; p value 0.001).

**Table 5:** Differences in BSI caused by Gram positive and Gram negative bacteria, and between MDRs and non-MDRs

Variables	Number	Antibiotic resistance			
		MDRs (n = 40)	Non-MDRs (n = 49)	Odds ratio (95 % CI)	p value
*Univariate analysis*					
Male gender					
Yes	55	23	32	0.72 (0.31–1.69)	0.450
No	34	17	17		
Age (± SD)		24.05 ± 2.41	31.09 ± 3.32	0.21 (0.19–0.79)	<0.001
Paediatric patients					
Yes	58	20	38	0.29 (0.11–0.72)	0.007
No	31	20	11		
Cancer type					
Leukemia	32	14	18	0.93 (0.9–2.21)	0.862
Lymphoma	15	7	8	1.08 (0.35–3.30)	0.887
Myeloma	4	1	3	0.39 (0.04–3.93)	0.624
Solid cancer	10	4	6	0.79 (0.21–3.04)	1
Undiagnosed	26	12	14	1.07 (0.43–2.68)	0.887
Haematologic cancers					
Yes		31	23	3.89 (1.53–9.86)	0.003
No		9	26		
Febrile neutropenia	21	10	11	1.15 (0.43–3.07)	0.778
Others	68	30	38		
Year of infection					
2010	18	8	10	0.97 (0.34–2.76)	1
2011	29	16	15	1.51 (0.63–3.63)	0.353
2012	31	17	14	1.84 (0.76–4.46)	0.17
2013	11	5	6	1.02 (0.29–3.63)	1
Patient location					
Child health	46	20	26	0.86 (0.38–2.04)	0.778
Haematology	25	12	13	1.18 (0.47–2.99)	0.718
Others	15	7	8	1.08 (0.36–3.31)	0.887
CTX resistance					0.001
Yes	40	26	14	4.64 (1.89–11.39)	
No	49	14	35		
Gram negatives					
Yes	49	34	15	12.8 (4.45–37.05)	<0.001
No	40	6	34		
*Enterobactericeae*					
Yes	32	28	4	26.2 (7.70–89.44)	<0.001
No	57	12	45		
Pseudomonads					
Yes	10	0	10	–	0.004
No	79	40	39		
Multivariate factor(s)					
	*Enterobacteriaceae*	33.6 (6.41–88.73)	<0.001		

Odds ratio in multivariate analysis were adjusted for age, paediatric patients, cefotaxime resistance, Gram-negative bacteria, *Enterobacteriaceae* and Pseudomonads

*MDRs* multidrug resistant strains, *CI* confidence interval, *SD* standard deviation, *CTX* cefotaxime

## Discussion

To our knowledge, this is the first study conducted in Ghana to document the microbiological features of bacteraemia among oncology patients. This retrospective study disclosed several important findings. Blood stream infections accounted for 93 of 453 blood cultures from cancer patients. Three observations merit attention. First, *Staphylococcus epidermidis* was the most frequently isolated organism (n = 28/93, 30.1 %) at an average rate of 7 isolates per year. Second, the frequencies of BSI pathogens during the entire period of observation from 2010 through 2013 did not change significantly. Infections by GNB have predominated in such cancer patients in several reported studies with comparable patient populations [18–20]. However, the aetiology of BSI in cancer patients is dramatically changing with clear shifts in infecting organisms; so that now BSI with single pathogens are due largely to Gram-positive cocci [1, 7, 21]. There are no previous surveys in Ghana to compare our data; and the reasons why GPB and GNB are dominant in our institution over the last 4 years remain unknown. Last, about 60 % of the *Entrobacteriaceae* were cefotaxime resistant. We are unable to determine if the isolates were positive for beta-lactamases, which limits the scope for comparing results from this study with existing literature. However, given that the variety of class C cephalosporinases (AmpCs) and extended spectrum beta-lactamases (ESBLs) constitute the predominant mechanisms for cephalosporin resistant worldwide [9, 10, 22], the high levels of cefotaxime-resistance observed in our study may be compatible with findings from other works in Ghana reporting high prevalence of ESBLs (>45 %) in the family *Enterobacteriaceae* [9, 23]. Carbapenems are often the treatment of choice for AmpC and ESBL-producing organisms. However, susceptibility data to carbapenems were unavailable; and we are unable to determine if cefotaxime-resistant *Enterobacteriaceae* reported in this study comprised isolates resistant to carbapenems.

Amoxicilin plus clavulanic acid, cefotaxime, gentamicin and ciprofloxacin are the predominant empiric therapies for treatment of blood stream infections in Ghana. Hovewer, the general antibiogram of the GNB and GPB revealed an overall high resistance to many of the routinely used drugs. It is the experience in KBTH that resistance to many routinely used antibiotics is high; and the prevalence is rising [9, 10]. We therefore believe that the choice of empiric therapy should vary according to locally prevalent isolates and their resistance patterns. Currently, use of cefotaxime or amoxicillin plus clavulanic acid as empirical monotherapy for febrile illness in cancer patients across most centers may not be justified due to the high levels of in vitro resistance observed against these drugs. Amikacin and levofloxacin were the most active antibiotics against GNB; and represent better alternatives over gentamicin and ciprofloxacin. Levofloxacin and amikacin have been on the Ghanaian market for a relatively short period of time since 2002; amikacin is parenteral and less likely to be misused or abused. Moreover, these antibiotics are expensive and usually prescribed for more serious infections, although systemic therapy over a period may also favour the selection of levofloxacin or amikacin resistant strains. In this study, *Staphylococcus aureus* remained fully susceptible to oxacillin, reflecting the low prevalence of methicillin-resistant staphylococci recently noted in KBTH [24]. Also, *Staphylococcusepidermidis* were the predomiant organisms, and exhibited high susceptibility to ampicillin. Given the low levels of polymicrobial infections, and the dual dominance of BSI by GPB and GNB, perhaps the combination therapy of amikacin plus a penicillinase stable penicillin such as cloxacillin would offer good empirical coverage across the different bacteria groups.

Multidrug resistant phenotypes accounted for 15.0 % (n = 6/40) of Gram-positive organisms, and were mostly vancomycin-resistant *Staphylococcusepidermidis* (n = 2/6) and vancomycin-resistant *Streptococcus* species (n = 2/6). Our concern regarding the MDR isolates of GPB is twofold. To begin with, *S. epidermidis* and other coagulase-negative staphylococci isolated from blood cultures were previously often considered contaminants. In this study, *S. epidermidis* was the leading cause of BSI in both adults and paediatric cancer patients. Second, the presence of *S*. *epidermidis* resistant to vancomycin is also worrying. The occurrence of MDR vancomycin-resistant *S. epidermidis* and *Streptococcus* species, although rare, presupposes that vancomycin may not be a completely reliable antibiotic in the empiric treatment of infections due to Gram positive cocci, and underpins the importance of routine antibiogram to the management of infections in our institution.

We evaluated patient characteristics associated with aetiology of BSI infections in cancer patients. Notably, paediatric cancer patients were less often infected with Gram-negative organisms. Our results are consistent with previously demonstrated findings in which Gram-positive cocci were the predominant cause of BSI in pediatric cancer patients in Groningen and Amsterdam (the Netherlands) and in Bern (Switzerland) [21]. Similarly, in a previous report from the National Cancer Institute at Cairo University, Gram-positive cocci were found to account for 61.9 % of the total isolates from paediatric cancer patients [25]. Recently, many workers have indicated that the prominence of Gram-positive organisms in paediatric cancer patients may reflect a more widespread chemotherapy-induced mucositis and the presence of indwelling intravascular devices [7, 26, 27].

There are some potential limitations of this study that should be discussed briefly. Blood stream infections in cancer patients over the 4-years were identified as including 453 patients. Whereas the limited number may reflect the relative incidence of organisms in a Ghanaian tertiary care setting biased by its referral policies, it also highlights a decline in the availability of patients’ specimens for institutional research and public health surveillance due to a marked increase in commercial microbiology test services. Note also that by being retrospective, susceptibility testing on all isolates were not available for many of the antibiotics. Also patients had been stratified with predetermined definitions to which we were unable to fully assess clinical history for correlations that might contribute to risk factor analysis. It is worth noting that oxacillin susceptibility (rather than cefoxitin) was used in reporting MRSAs [16]. The occurrence of MRSAs in this study may therefore be underestimated. Despite these shortcomings, our findings offer information that should help to inform the suitability of local policies in managing BSI in cancer patients.

In conclusion, our data highlights a current trend of GNB/GPB duoplay in BSI with a clear stratification of risk factors for aetiologic agents—whereas paediatric patients were at increased risk of Gram positive bacteraemia, adults were significantly associated with Gram-negative organisms with considerable multidrug resistance phenotypes among *Enterobacteriaceae*. Adapting selective antimicrobial use based on local epidemiologic data will maximize clinical outcome of empiric antibiotic therapy among cancer patients in our institution. Our findings should compel a concerted effort in our institution and elsewhere for local monitoring of bacterial isolates in order to adapt policies on antibiotic therapy based on the local antibiotic susceptibility profiles.

## Abbreviations

µg: Microgram
ANOVA: Analysis of variance
ATCC: American Type Culture Collection
BSI: Blood stream infections
CI: Confidence interval
CLSI: Clinical and Laboratory Standards Institute
CTX: Cefotaxime
ESD: Eric Sampene-Donkor
MEA: Michael Ebo Acquah
GNB: Gram-negative bacteria
GPB: Gram positive bacteria
KBTH: Korle-Bu Teaching Hospital
LAK: Labi Appiah-Korang
MDR: Multidrug resistance
NON: Noah Obeng-Nkrumah
NJ: New Jersey
OR: Odds ratio
r: Pearson product-moment correlation coefficient
ROC: Receiver operating characteristic
SD: Standard deviation
SPSS: Statistical Package for Social Sciences
r_s_: Spearman’s rank correlation coefficient
USA: United States of America
WHO: World Health Organisation
